# Decreased diffusion tensor image analysis along the perivascular space index is related with clinical classification in essential tremor

**DOI:** 10.1093/braincomms/fcaf240

**Published:** 2025-06-17

**Authors:** Yan Qin, Runcheng He, Xun Zhou, Mingqiang Li, Na Tan, Lanqing Liu, Yuwen Zhao, Zhenhua Liu, Qian Xu, Jifeng Guo, Xinxiang Yan, Beisha Tang, Dongcui Wang, Qiying Sun

**Affiliations:** Department of Radiology, Xiangya Hospital, Central South University, Changsha, Hunan 410008, China; National Clinical Research Center for Geriatric Disorders, Xiangya Hospital, Central South University, Changsha, Hunan 410008, China; Department of Neurology, The Second Xiangya Hospital, Central South University, Changsha, Hunan 410011, China; Department of Neurology, Xiangya Hospital, Central South University, Changsha, Hunan 410008, China; Department of Geriatric Neurology, Xiangya Hospital, Central South University, Changsha, Hunan 410008, China; Department of Neurology, The First Affiliated Hospital of University of South China, Hengyang, Hunan 421001, China; Department of Ultrasound Medicine, Shenzhen Yan Tian District People's Hospital (Group), Shenzhen, Guangdong 518083, China; Department of Geriatric Neurology, Xiangya Hospital, Central South University, Changsha, Hunan 410008, China; National Clinical Research Center for Geriatric Disorders, Xiangya Hospital, Central South University, Changsha, Hunan 410008, China; Department of Neurology, Xiangya Hospital, Central South University, Changsha, Hunan 410008, China; National Clinical Research Center for Geriatric Disorders, Xiangya Hospital, Central South University, Changsha, Hunan 410008, China; Department of Neurology, Xiangya Hospital, Central South University, Changsha, Hunan 410008, China; National Clinical Research Center for Geriatric Disorders, Xiangya Hospital, Central South University, Changsha, Hunan 410008, China; Department of Neurology, Xiangya Hospital, Central South University, Changsha, Hunan 410008, China; National Clinical Research Center for Geriatric Disorders, Xiangya Hospital, Central South University, Changsha, Hunan 410008, China; Department of Neurology, Xiangya Hospital, Central South University, Changsha, Hunan 410008, China; National Clinical Research Center for Geriatric Disorders, Xiangya Hospital, Central South University, Changsha, Hunan 410008, China; Department of Neurology, Xiangya Hospital, Central South University, Changsha, Hunan 410008, China; National Clinical Research Center for Geriatric Disorders, Xiangya Hospital, Central South University, Changsha, Hunan 410008, China; Department of Neurology, Xiangya Hospital, Central South University, Changsha, Hunan 410008, China; Department of Radiology, Xiangya Hospital, Central South University, Changsha, Hunan 410008, China; National Clinical Research Center for Geriatric Disorders, Xiangya Hospital, Central South University, Changsha, Hunan 410008, China; National Clinical Research Center for Geriatric Disorders, Xiangya Hospital, Central South University, Changsha, Hunan 410008, China; Department of Geriatric Neurology, Xiangya Hospital, Central South University, Changsha, Hunan 410008, China

**Keywords:** essential tremor, diffusion tensor imaging along the perivascular space (DTI-ALPS) index, glymphatic function, clinical classification

## Abstract

The glymphatic dysfunction involved in various neurodegenerative diseases. However, the relationship between glymphatic activity and essential tremor (ET) has not been fully elucidated. Our study explored the impact of glymphatic function on ET and its clinical classification. Participants comprised 37 pure ET, 38 ET-plus and 50 normal controls. Glymphatic function was evaluated via the diffusion tensor image analysis along the perivascular space (DTI-ALPS) index. Statistical comparisons of DTI-ALPS index among patients with pure ET, those with ET-plus and normal controls were conducted using general linear model analysis. Age, gender and disease duration were included as confounding variable. To confirm the relation between the DTI-ALPS index and the clinical characteristics of pure ET and ET-plus, we conducted partial Spearman rank correlation analyses while controlling for age and disease duration. The DTI-ALPS index in ET-plus patients was significantly lower than that in normal controls (*P* = 0.004) and pure ET patients (*P* = 0.010). In ET-plus patients, the DTI-ALPS index demonstrated a significant inverse relationship with disease duration (*r* = −0.330, *P* = 0.043). No significant correlations were found between the DTI-ALPS index and clinical severity of ET (all *P* > 0.05). We have identified for the first time that DTI-ALPS index could be used as a potential biomarker for the clinical classification of ET. The DTI-ALPS index was intimately correlated to disease duration in ET-plus patients.

## Teaser

Qin *et al.* report that the diffusion tensor imaging along the perivascular space index is significantly lower in essential tremor plus than in pure essential tremor and negatively correlates with disease duration in essential tremor plus. This work suggests its potential as a biomarker for clinical classification in essential tremor.

## Graphical Abstract

**Graphical Abstract:**
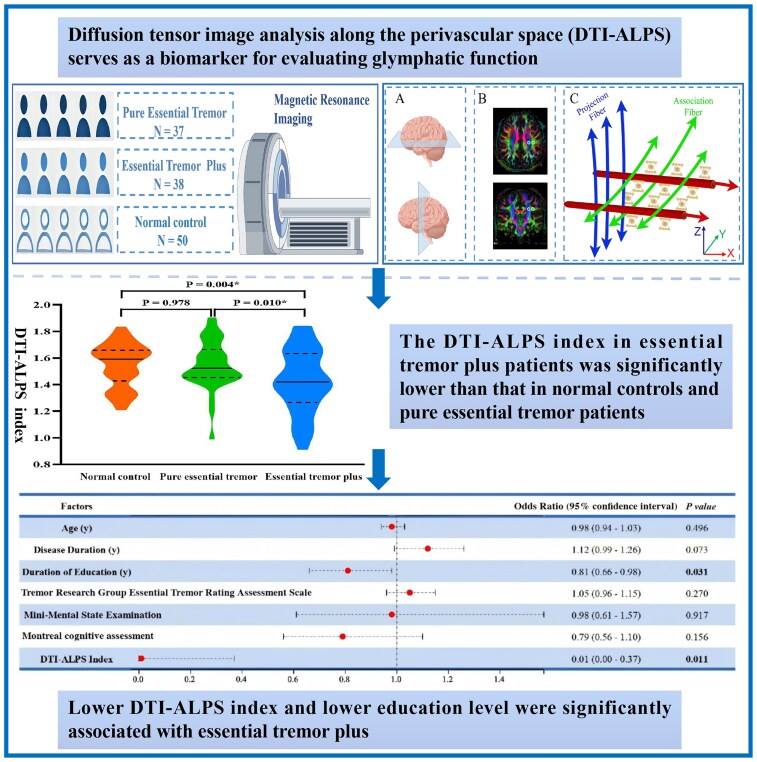

## Introduction

Essential tremor (ET), with a global prevalence nearing 0.9%, ranks among the most prevalent movement disorders.^1^ Recent years have witnessed significant evolution in our understanding of this disease. According to the 2018 International Parkinson and Movement Disorder Society (IPMDS) consensus guidelines on tremor disorders, ET is characterized by bilateral upper limb action tremor persisting for a minimum of three years. This condition may also involve tremors in additional body regions, such as the head, face, voice or lower extremities.^2^ The 2018 consensus statement introduced the term ET-plus, describing ET accompanied by additional ‘soft neurological signs’ of unclear clinical relevance—such as mild memory impairment, questionable dystonic posturing, impaired tandem gait or other mild neurological signs of unknown significance.^2^ This updated diagnostic framework has sparked both interest and controversy among researchers and clinicians.^3^ ET exhibits significant clinical heterogeneity.^4^ The pathophysiology of ET and the exact mechanisms that affects the clinical classification of ET are still not entirely understood. In the absence of clear objective biomarkers, it is relatively challenging to clinically differentiate pure ET and ET-plus based solely on clinical presentations. Thus, the search for new markers is highly necessary.

Recent studies have identified a ‘neurotoxin clearance’ system in the brain, referred to as the glymphatic system. This system operates through the dynamic exchange between cerebrospinal fluid (CSF) and interstitial fluid (ISF) within perivascular space, facilitating the removal of metabolic products and neurotoxic waste.^5,6^ Disruptions to any component of the glymphatic pathway, such as reduced CSF influx, altered CSF–ISF interaction or compromised ISF drainage, may adversely affect glymphatic functionality.^7^ Animal researches have indicated that impaired glymphatic clearance contributes to the pathological aggregation of amyloid-β and tau proteins.^8-10^ Previous studies have established the association between glymphatic system impairment and multiple neurodegenerative disorders such as Alzheimer’s disease and Parkinson’s disease.^11^ However, its role in ET remains unelucidated. Diffusion tensor image analysis along the perivascular space (DTI-ALPS), as a non-invasive method, has been introduced to investigate the glymphatic system in 2017.^12^ Subsequently, this advanced method was applied in studies of various diseases including Alzheimer’s disease, Parkinson’s disease and neuromyelitis optic spectrum disorder. The DTI-ALPS index, derived from diffusion tensor imaging (DTI), quantifies water diffusion dynamics by assessing the ratio of perivascular space (PVS) directional diffusion to interstitial free water diffusion. Studies confirm its utility in evaluating bulk water movement along the PVS.^12,13^

The objective of our study was to assess glymphatic system activity differences among pure ET patients, ET-plus patients and normal control (NC) subjects by application of DTI-ALPS index, to explore the effects of glymphatic function on ET and its clinical classifications. Furthermore, we aimed to examine potential correlations between the DTI-ALPS index and disease severity.

This study aimed to assess glymphatic system activity differences among normal controls (NC), pure essential tremor (ET) patients and ET-plus cases using the DTI-ALPS index. We sought to investigate how glymphatic function influences ET and its clinical subtypes, as well as examine potential correlations between the DTI-ALPS index and disease severity.

## Materials and methods

### Patients

Our study enrolled 75 patients with essential tremor (ET), comprising 37 cases of pure ET and 38 cases of ET-plus, who were consecutively recruited from both inpatient and outpatient clinics. Additionally, 50 age- and sex-matched NC were recruited from the health examination center. The recruitment period spanned from 1 May 2021 to 30 December 2022, and was conducted through the Parkinson’s Disease & Movement Disorders Multicenter Database and Collaborative Network in China (http://pd-mdcnc.com). All participants provided written informed consent. NC exhibited no neurological abnormalities upon clinical examination. They were free of any history of neurological disorders, psychiatric conditions or major systemic illnesses. In accordance with the standardized criteria established by the 2018 consensus statement, each patient's diagnosis was independently confirmed by two senior neurologists. Participants were excluded if they met any of the following conditions: (i) exhibited tremor linked to other central nervous system disease, such as Parkinson’s disease, dystonic tremor and stroke; (ii) presented with orthostatic tremor, isolated vocal tremor and task-specific tremor; (iii) with pathogenic short tandem repeats in tremor-associated genes, including *FMR1, SADM12, PPP2R2B, NOP56, ATXN1, ATXN2, ATXN3, CACNA1A, ATXN7, ATXN8OS, ATXN10, DAB1, BEAN1, TBP* and *ATN1*^14^; (iv) were unable to finish the MRI protocol (claustrophobia, MR-unsafe implants, pregnancy or breastfeeding) and (v) had DTI data of insufficient quality for calculating the ALPS index. This research protocol was reviewed and approved by the Medical Ethics Committee of the Xiangya Hospital, Central South University, with conduct strictly adhering to the Declaration of Helsinki ethical standards.

### Clinical assessments

Demographic and clinical characteristics were systematically recorded in the study database. All participants received standardized neurological and neuropsychological evaluations administered by two board-certified neurologists. Tremor severity was assessed using the Tremor Research Group Essential Tremor Rating Assessment Scale (TETRAS),^15^ with its subcomponents applied as follows: TETRAS-I (0–4 points) quantified tremor-induced functional limitations in daily activities; TETRAS-II documented tremor localization patterns and tremor severity. Non-Motor Symptoms Scale (NMSS) measured the severity of non-motor symptoms, while cognitive function was screened through the Mini-Mental State Examination (MMSE) and Montreal Cognitive Assessment (MoCA).^16^

ET-plus diagnosis adhered to the new consensus criteria, characterized by ET coexisting with any of the following neurological soft signs: (i) impaired tandem gait (≥2 missteps out of a 10-step trial); (ii) mild cognitive impairment [determined as MMSE <17 (illiterate), <20 (elementary education) or <24 (≥middle school education or above); or MoCA <26 (<25 if education ≤12 years)]; (iii) questionable dystonic posturing (Unified Dystonia Rating Scale score ≥1); (iv) resting tremor (observed in standardized positions: standing with relaxed arms or seated with forearm support)^4^ and (v) questionable myotonia.

### MRI acquisition

All participants were subjected to standardized MRI protocols conducted on a 3.0-T Siemens Healthcare scanner (Germany) with integrated 64-channel head-neck phased-array coils. Identical scanning protocols were implemented across subjects, incorporating fluid-attenuated inversion recovery (FLAIR) and T2-weighted sequences to rule out structural brain abnormalities. DTI was acquisitioned using axial spin-echo single-shot echo-planar pulse sequences with the following parameters: repetition time/echo time = 4200/68 ms, flip angle = 90°, slice thickness = 2.00 mm, number of slices = 70, voxel size = 2.0 ×2.0 × 2.0 mm^3^, field of view= 264 × 264 mm^2^ and *b*-value = 1000 s/mm^2^ applied along 64 non-collinear directions.

### Diffusion tensor image analysis along the perivascular space index calculation

**Figure 1:**
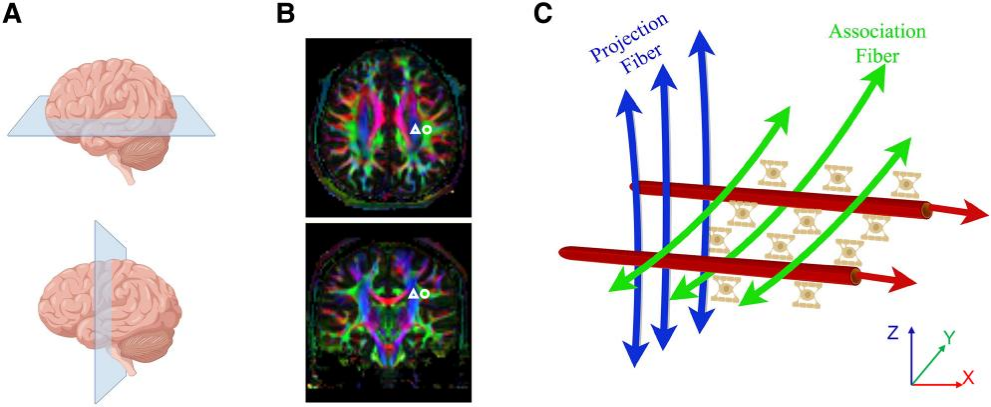
**Schematic drawing of the principle underlying the diffusion tensor imaging analysis along the perivascular space (DTI-ALPS)**. (**A**) indicates a schematic diagram of the selected planes, with the upper image representing an axial plane at the lateral ventricle body level, and the lower image showing a coronal plane at the lateral ventricle body level. (**B**) Left-hemisphere regions of interest positioned on the fractional anisotropy map (triangles: projection fibre; circles: association fibre). (**C**) Perivascular space perpendicular to projection and association neural fibres. Therefore, diffusivity along the *x*-axis at these areas mostly reflects total or macroscopic movement of water in the direction of the perivascular space direction.

Initial quality assessment of DTI scans was performed via visual inspection to identify potential artefacts. Subsequent preprocessing and tensor model fitting were conducted using the FMRIB Software Library (FSL, version 6.0.1, http://www.fmrib.ox.ac.uk/fsl/). The preprocessing pipeline consisted of three sequential steps: correction for eddy currents, correction for motion artefacts and skull stripping using the Brain Extraction Tool. Tensor model fitting generated multiple diffusion parametric maps, including the principal eigenvector map (V1), fractional anisotropy (FA) and directional diffusivity maps (D_xx_, D_yy_ and D_zz_).^11^ Two board-certified neuroradiologists (Y.Q. and D.W. with >5 years of experience) independently placed 5-mm spherical regions of interest (ROI) within the projection and association fibre regions at the level of the lateral ventricular body (Fig. 1). This placement was guided by visualization of the V1-modulated FA map using the FSLEYES module. Mean diffusivity values were subsequently extracted from the corresponding directional diffusivity maps. The computational analysis incorporated four key diffusion parameters: *x*-axis diffusivity in both projection (D_xx, proj_) and association fibres (D_xx, assoc_), along with *y*-axis diffusivity in projection fibres (D_yy, proj_) and *z*-axis diffusivity in association fibres (D_zz, assoc_). These variables were then incorporated into the established DTI-ALPS index formula for quantitative assessment.^12^ The DTI-ALPS index = mean(D_xx, proj_, D_xx, assoc_)/mean(D_yy, proj_, D_zz, assoc_). Y.Q. and D.W. measured the DTI-ALPS index independently with each other, blinded to the diagnosis.

Given the potential confounding effects of white matter hyperintensities (WMHs) on DTI-ALPS measurements, detailed WMH characterization was performed. FLAIR sequence images (repetition time/echo time/inversion time, 9000/83/2500 ms; section thickness 5 mm) were analysed using a standardized protocol. The Scheltens semiquantitative visual rating system was applied to independently assess two distinct categories: (i) periventricular WMHs and (ii) lobar WMHs.^17^ To minimize the confounding effects of severe white matter lesions on our analysis, we excluded patients with a periventricular WMH score greater than 3 or a lobar WMH score greater than 3 from our study.

### Statistical analysis

Continuous variables following normal distribution were expressed as mean ± standard deviation, whereas non-normally distributed data were described using the median (25th percentile, 75th percentile). Categorical variables were summarized using percentages and frequency counts. Demographic differences across pure ET, ET-plus and NC groups were analysed through Kruskal–Wallis tests with Bonferroni correction. The Bonferroni-adjusted significance level was set at *P* < 0.017 (0.05/3). A binary logistic regression model incorporating age, disease duration, duration of education, TETRAS-II scores, MMSE scores and MoCA scores, and the DTI-ALPS index was constructed to identify determinants of ET-plus diagnosis. We utilized a general linear model to compare clinical and neuroimaging characteristics among the groups. Age, gender and disease duration were included as confounding variables. To control for multiple testing, the *P*-values were corrected using the Bonferroni adjustment. Furthermore, Spearman’s correlation was employed to analyse potential links between the DTI-ALPS index and both age and disease duration. Controlling for age and age at onset, the correlations between the DTI-ALPS index and the scores of various clinical scales were assessed using Pearson's partial correlation analysis. Statistical significance was set at *P* < 0.05 (two-tailed). Statistical significance was defined as a two-tailed *P*-value of <0.05. Inter-rater reliability of DTI-ALPS was examined by intraclass correlation coefficient (ICC). Data analysis was conducted using SPSS (Version 24.0, IBM Corp.).

## Results

### Demographics and clinical characteristics

In total, 37 pure ET (21 males; mean age: 47.08 ± 11.85 years), 38 ET-plus (15 men; mean age: 54.26 ± 15.91 years) and 50 NC (23 males; mean age: 51.78 ± 8.19 years) were included. No statistically significant variations were observed across the three groups regarding demographic characteristics (gender distribution), lifestyle factors (smoking habits and alcohol intake), comorbid conditions (hypertension, diabetes and hyperlipidaemia) or cognitive assessment scores (MMSE scores) (all *P* > 0.05). The mean age at enrolment was significantly higher in the ET-plus patients compared to pure ET patients (*P* = 0.012). However, no significant difference in AAO was observed between pure ET and ET-plus patients (*P* = 0.219). Additionally, periventricular WMH scores and lobar WMH scores showed no statistically significant variations across the three patient groups. The characteristics of the participants were summarized in Table 1. In the ET-plus group, mild cognitive impairment was observed in 13 patients (34.2%), while an equal number exhibited impaired tandem gait (34.2%). Additionally, 12 patients (31.6%) presented with questionable dystonic posturing, 5 (13.2%) displayed resting tremor and 15 (39.5%) showed multiple soft neurological signs. Patients with ET-plus exhibited significantly higher TETRAS-I scores compared to those with pure ET (18.16 ± 7.98 versus 14.38 ± 6.40, *P* = 0.022) after correction for age and gender. However, no significant differences were observed in TETRAS-I (10.35 ± 6.55 versus 12.82 ± 9.88, *P* = 0.198) or NMSS scores (4.62 ± 6.51 versus 7.58 ± 8.80, *P* = 0.094) between the two subgroups.

**Table 1:** Demographic and clinical characteristics of the study participants

	NC (*n* = 50)	Pure ET subtype (*n* = 37)	ET-plus subtype (*n* = 38)	*P-*values	
Male, *n* (%)	23 (46.0%)	21 (56.8%)	15 (39.5%)	0.317^a^	
Age (y)	52 (48,55.5)	50 (39,54)	58 (42.25,66.75)	**0**.**001**^b^	***p*** ^1^ **<** **0.001**^c^
					*P* ^2^ = 0.803^c^
					***P*** ^3^ **=** **0.012**^c^
Duration of Education (y)		15 (12,16)	9 (6,12)	**<0**.**001**^c^	
AAO (y)		43 (32,50)	49.5 (32,54.75)	0.219^c^	
Duration (y)		5 (3,7)	6.5 (4,11.75)	**0**.**036**^c^	
Smoking		7 (18.9%)	7 (18.4%)	0.956^a^	
Alcohol consumption, *n* (%)		7 (18.9%)	6 (15.8%)	0.720^a^	
Hypertension, *n* (%)	12 (24.0%)	7 (18.9%)	10 (26.3%)	0.739^a^	
Diabetes, *n* (%)	5 (10.0%)	3 (8.1%)	4 (10.5%)	0.932^a^	
Hyperlipidaemia, *n* (%)	9 (18.0%)	6 (16.2%)	9 (23.7%)	0.687^a^	
MMSE	28.28 ± 1.55	28.70 ± 1.51	27.39 ± 2.92	0.073^b^	
MoCA	28.00 ± 1.43	27.51 ± 1.76	24.66 ± 4.30	**<0**.**001**^d^	*p* ^1^ = 0.257^c^
					***P*** ^2^ **< 0.001**^c^
					***P*** ^3^ **=** **0.003**^c^
TETRAS part I		10.35 ± 6.55	12.82 ± 9.88	0.198^d^	
TETRAS part II		14.38 ± 6.40	18.16 ± 7.98	**0**.**022**^d^	
NMSS		4.62 ± 6.51	7.58 ± 8.80	0.094^d^	
Periventricular WMH scores	0.5 (0,1)	0 (0,1)	1 (0,2)	0.084^b^	
Lobar WMH scores	0.5 (0,1)	0 (0,3)	1 (0,2.75)	0.191^b^	
D_xx, proj_ (×10^−3^mm^2^/s)	0.63 (0.60, 0.65)	0.62 (0.60, 0.64)	0.59 (0.56, 0.63)	**0**.**023**^b^	*p* ^1^ = 0.637^c^
					***P*** ^2^ **=** **0.009**^c^
					*P* ^3^ = 0.037^c^
D_xx, assoc_ (×10^−3^mm^2^/s)	0.60 (0.56, 0.66)	0.61 (0.58, 0.65)	0.63 (0.57, 0.67)	0.403^b^	
D_yy, proj_ (×10^−3^mm^2^/s)	0.49 (0.43, 0.53)	0.48 (0.44, 0.54)	0.52 (0.49, 0.56)	**0**.**010**^b^	*p* ^1^ = 0.942^c^
					***P*** ^2^ **=** **0.005**^c^
					***P*** ^3^ **=** **0.015**^c^
D_zz, assoc_ (×10^−3^mm^2^/s)	0.31 (0.29, 0.34)	0.30 (0.29, 0.34)	0.33 (0.29, 0.36)	0.219^b^	

^a^ *P*: Chi-squared test.

^b^ *P*: Kruskal–Wallis test.

^c^ *P*: Mann–Whitney U-test.

^d^ *P*:General linear model, age, gender and disease duration were included as confounding variables.

*P* ^1^: the *P-*value between the pure essential tremor and normal control group.

*P* ^2^: the *P-*value between the essential tremor plus and normal control group.

*P* ^3^: the *P*-value between the pure essential tremor and essential tremor plus group. The *P*-value was set at 0.017 (0.05/3) after Bonferroni correction with three multiple corrections. Significant *P*-values are in bold.

ET, essential tremor; NC, normal control; BMI, Body mass index; AAO, age at onset; TETRAS, Tremor Research Group Essential Tremor Rating Assessment Scale; NMSS, Non-Motor Symptom assessment scale; MMSE, Mini-Mental State Examination; MoCA, Montreal cognitive assessment; WMHs, white matter hyperintensities; D_xx, proj_, diffusivity along the *x*-axis in the projection fibre area; D_xx, assoc_, diffusivity along the *x*-axis in the association fibre area; D_yy, proj_, diffusivity along the *y*-axis in the projection fibre area; D_zz, assoc_, diffusivity along the *z*-axis in the association fibre area.

### Comparison of the DTI-ALPS index between the three groups

**Figure 2:**
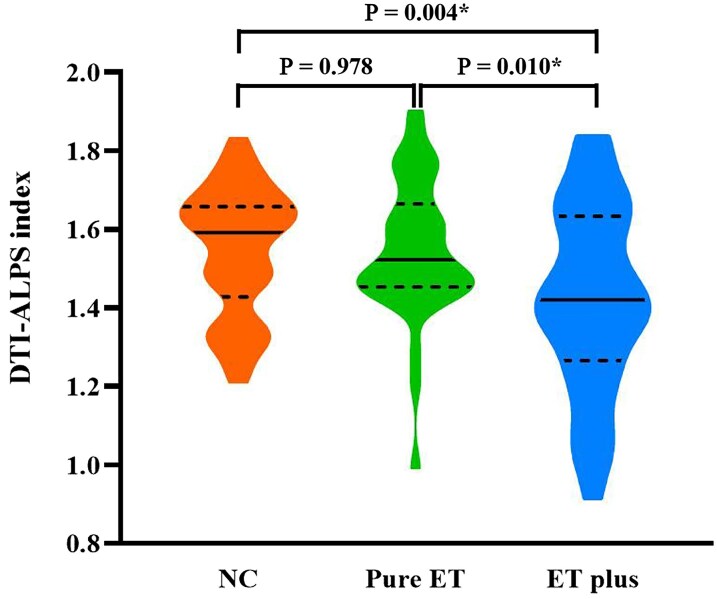
**Violin plots of DTI-ALPS indices for normal controls (*n* = 50), pure ET (*n* = 37) and ET-plus (*n* = 38).** Dotted lines indicate the interquartile range [75th (upper horizontal line) and 25th (lower horizontal line)]. Full line indicates the median. The figure shows *P*-values after adjusting for age and gender using a general linear model with Bonferroni correction. *Significant to *P* < 0.05. DTI-ALPS index, diffusion tensor imaging along the perivascular space.

The ICC was 0.857 (*P* = 0.013). The median DTI-ALPS indexes were 1.59 (1.45, 1.65) in the NC group, 1.52 (1.46, 1.64) in the pure ET group and 1.42 (1.28, 1.62) in the ET-plus group. Adjusted for gender, age and disease duration in general linear model, the DTI-ALPS index was significantly reduced in the ET-plus subtype compared to both the pure ET subtype (*P* = 0.010) and NC group (*P* = 0.004). The pure ET subtype showed no statistically significant difference in DTI-ALPS index compared to the NC group (*P* = 0.978) (Fig. 2).

### Association between the DTI-ALPS index and clinical phenotypes of ET

**Figure 3:**
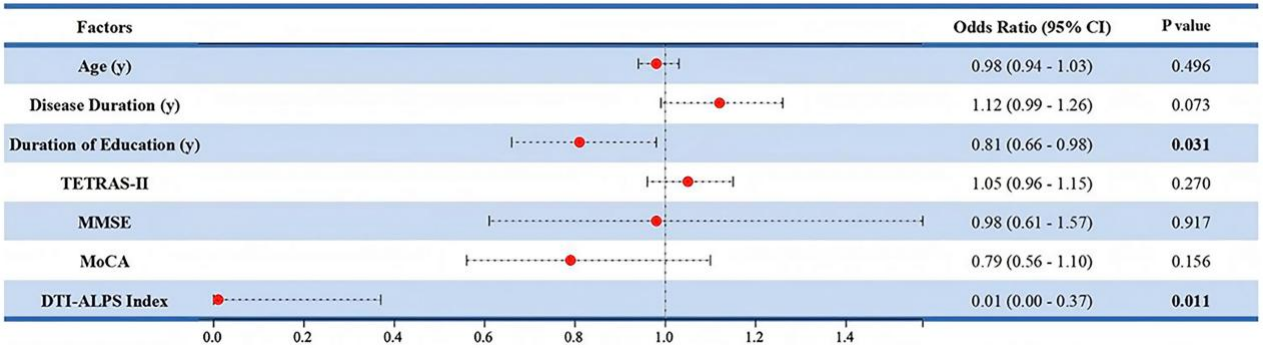
**Binary logistic regression analysis of factors related to ET-plus.** The horizontal line segments represent the confidence intervals of the odds ratio for each independent variable, with the central dot indicating the odds ratio value (*n* = 75). TETRAS, the Tremor Research Group Essential Tremor Rating Assessment Scale; MMSE, Mini-Mental State Examination; MoCA, Montreal cognitive assessment; DTI-ALPS index, diffusion tensor imaging along the perivascular space; OR, odds ratios; CI, confidence intervals. Values in bold refer to statistically significant differences (*P* < 0.05).

According to binary logistic regression analysis, a lower education level [odds ratio (OR) = 0.81, 95% confidence interval (CI): 0.66–0.98, *P* = 0.031)] and a reduced DTI-ALPS index (OR = 0.01, 95% CI: 0.00–0.37, *P* = 0.011) showed significant associations with ET-plus (Fig. 3).

### Association between DTI-ALPS indexes and clinical characteristics in ET patients

In the overall ET cohort, the DTI-ALPS index exhibited a significant inverse relationship with both age (*r* = −0.234, *P* = 0.043) and disease duration (*r* = −0.293, *P* = 0.011). Within the ET-plus subgroup, disease duration demonstrated a notable negative correlation with the DTI-ALPS index (*r* = −0.330, *P* = 0.043). Conversely, in the pure ET subgroup, neither age (*r* = −0.173, *P* = 0.306) nor disease duration (*r* = 0.064, *P* = 0.707) showed a statistically significant association with the DTI-ALPS index.

After adjusting for age and disease duration, partial correlation analysis revealed no significant relationship between the DTI-ALPS index and TETRAS-I scores, TETRAS-II scores, NMSS scores, MMSE scores and MoCA scores in either the ET-plus or pure ET subgroups (all *P* > 0.05) (Table 2).

**Table 2:** Correlations of DTI-ALPS indexes with motor and non-motor symptoms in pure ET and ET-plus

	Pure ET		ET-plus	
	Correlation coefficient	*P*-values	Correlation coefficient	*P*-values
TETRAS-I scores	−0.015	0.938	0.009	0.960
TETRAS-II scores	0.044	0.822	0.037	0.830
NMSS scores	−0.037	0.834	0.171	0.173
MMSE scores	−0.045	0.818	−0.105	0.542
MoCA scores	−0.140	0.468	−0.232	0.173

Note: Adjusted for age and disease duration. ET, Essential tremor; TETRAS, the Tremor Research Group Essential Tremor Rating Assessment Scale; NMSS, Non-Motor Symptoms Scale; MMSE, Mini-Mental State Examination; MoCA, Montreal cognitive assessment.

A simple linear regression analysis was conducted for each clinical variable—gender, age, age at onset, disease duration, duration of education, TETRAS-I scores, TETRAS-II scores, MMSE scores, MoCA scores and NMSS scores—to assess their potential association with the DTI-ALPS index in patients with pure ET or ET-plus. Disease duration in ET-plus was the only variable that had significantly associated with DTI-ALPS index (β = −0.007, 95% CI: −0.140 to −0.000, *P* = 0.043).

## Discussion

In our study, we investigated the associations of glymphatic pathway with clinical classifications of ET and its clinical symptoms using the DTI-ALPS approach. Our findings revealed that impaired glymphatic activity, as indicated by a reduced DTI-ALPS index, showed a significant correlation with ET-plus. Additionally, disease duration was strongly linked to glymphatic dysfunction. These results further support the growing evidence implicating glymphatic impairment in ET-plus patients.

DTI-ALPS index serves as a non-invasive imaging biomarker for assessing cerebral fluid dynamics, offering indirect insights into glymphatic system activity. Growing evidence supports its role as a biomarker for glymphatic dysfunction in various neurological conditions.^18^ To minimize the confounding effects of severe white matter lesions on our analysis, we excluded patients with a periventricular WMH score greater than 3 or a lobar WMH score greater than 3 from our study. This exclusion criterion was implemented because extensive WMHs in these regions could significantly alter the integrity of white matter tracts and potentially bias the DTI-ALPS index, which is sensitive to microstructural changes in the brain. The results of our study showed no statistically significant differences in periventricular WMH scores or lobar WMH scores among the three groups of patients. This finding suggests that the severity of WMHs was comparable across the groups, and thus, the observed differences in DTI-ALPS indices are unlikely to be confounded by variations in WMHs burden.

Nevertheless, it should be acknowledged that the DTI-ALPS index comes with some inherent limitations. While it provides valuable insights into perivascular fluid dynamics, it does not directly measure glymphatic activity and may be influenced by other factors. Therefore, future studies should aim to combine the DTI-ALPS index with other advanced imaging modalities to provide a more comprehensive and direct evaluation of glymphatic system functionality. The DTI-ALPS index may partially reflect glymphatic system activity.^12^ The glymphatic system can remove pathological proteins and iron deposition from the brain parenchyma.^5,9,19^ Glymphatic dysfunction may compromise the clearance of pathogenic proteins such as amyloid beta, α-synuclein and tau, resulting in the accumulation of these protein aggregates within the interstitial fluid and neurons.^20,21^ The deposition of metabolic waste could trigger neurotoxicity and disrupt both synaptic transmission and neuronal function, resulting in a cascade of pathological damage.

DTI-ALPS index has been utilized as a metric for evaluating glymphatic activity across various neurodegenerative diseases. Previous researches have suggested that patients with Parkinson’s disease, Alzheimer’s disease and isolated rapid eye movement sleep behaviour disorder exhibit compromised glymphatic function, as measured by the DTI-ALPS index.^11,22,23^ Additionally, comparative analyses have revealed a lower DTI-ALPS index in patients with Parkinson’s disease relative to those with ET.^13,24^ However, there was no statistically significant disparity in the DTI-ALPS index when comparing the ET and NC groups.^24^ Hence, several researchers postulated that Parkinson’s disease is an α-synucleinopathy disease, whereas ET may not share this characteristic. In the 2018 consensus statement, the notion of ‘ET-plus’ was established, characterizing essential tremor that co-occurs with other neurological signs whose significance remains uncertain.^2^ However, the underlying aetiology and nature of ET-plus remain unclear. Previous pathological studies have reported a higher occurrence of pathological Lewy bodies in the brains of ET patients compared to normal elderly individuals.^25-27^ The presence of pathological Lewy bodies seems to be closely associated with glymphatic dysfunction. Excitingly, our study for the first time reveals a significantly reduced ALPS index in the ET-plus patients relative to pure ET cases, while the DTI-ALPS index in pure ET patients did not exhibit any notable difference when compared to that of normal individuals. This emerging finding indicated that ET-plus pathophysiology may be closely related to glymphatic dysfunction whereas pure ET pathophysiology is not characterized by glymphatic dysfunction. Thus, we hypothesize that impaired glymphatic function may contribute to the accumulation of pathological Lewy bodies, thereby playing a role in the development of soft neurological signs. The DTI-ALPS index could potentially serve as an important biomarker for the clinical classification of ET. Our study also found that glymphatic activity was not associated with the severity of motor or non-motor symptoms in either pure ET or ET-plus patients. This finding suggested that while glymphatic activity may be partially involved in the pathogenesis of ET-plus, it is not involved in the regulation of disease severity.

Interestingly, our study revealed a negative correlation between disease duration and the DTI-ALPS index specifically in ET-plus patients, but not in those with pure ET. This suggests that glymphatic activity may play a significant role in the disease progression of ET-plus, whereas pure ET likely follows a distinct pathophysiological mechanism less dependent on glymphatic function. Further prospective studies are warranted to validate this hypothesis. However, no significant associations were observed between the DTI-ALPS index and any of the clinical variables in pure ET patients. In contrast, the significant positive correlation between disease duration and the DTI-ALPS index in ET-plus patients further underscores the potential involvement of glymphatic dysfunction in this subtype. These findings support the inference that impaired glymphatic function contributes substantially to disease progression in ET-plus.

The DTI-ALPS index showed significant positive associations with MMSE and MoCA scores in patients with Alzheimer’s disease, Parkinson’s disease and related disorders.^12,18,23,28^ However, our study found no significant correlation between the DTI-ALPS index and MMSE or MoCA scores among ET patients. This discrepancy may be explained by the predominantly normal cognitive function of our study cohort, as individuals with significant cognitive impairment were excluded. Consequently, the variations in MMSE and MoCA scores among ET patients in our cohort may have been too subtle to detect. Notably, previous studies primarily included individuals with differing severities of cognitive decline, leading to markedly reduced MMSE/MoCA scores relative to our study population.

The limitation of our study is the reliance on the DTI-ALPS method for indirectly evaluating glymphatic function, as this approach examines only a single anatomical level of the brain and may not fully capture true glymphatic activity. Furthermore, the relatively modest sample size could limit the broader applicability of our results. Therefore, it is crucial for future research to include larger sample sizes to confirm and extend the findings of this study.

## Conclusion

Our findings provide the first evidence that the DTI-ALPS index could function as a biomarker for the clinical classification of ET. Furthermore, the DTI-ALPS index was intimately correlated to disease duration in ET-plus patients.

## Data Availability

Data sets analysed during the current study are available on reasonable request. All data have been anonymized.
